# Predictors of neonatal abstinence syndrome in buprenorphine exposed newborn: can cord blood buprenorphine metabolite levels help?

**DOI:** 10.1186/s40064-016-2576-8

**Published:** 2016-06-23

**Authors:** Darshan Shah, Stacy Brown, Nick Hagemeier, Shimin Zheng, Amy Kyle, Jason Pryor, Nilesh Dankhara, Piyuesh Singh

**Affiliations:** Department of Pediatrics, Quillen College of Medicine, East Tennessee State University, Johnson City, TN USA; Department of Pharmaceutical Sciences, Bill Gatton College of Pharmacy, East Tennessee State University, Johnson City, TN USA; Department of Pharmacy Practice, Bill Gatton College of Pharmacy, East Tennessee State University, Johnson City, TN USA; College of Public Health, East Tennessee State University, Johnson City, TN USA; Neonatal Perinatal Medicine, Vanderbilt University Medical Center/Monroe Carell Children’s Hospital, 2200 Children’s Way, Nashville, TN 37232 USA

**Keywords:** Buprenorphine, Neonatal abstinence syndrome, NAS, Cord blood, Metabolites

## Abstract

**Background:**

Buprenorphine is a semi-synthetic opioid used for the treatment of opioid dependence. Opioid use, including buprenorphine, has been increasing in recent years, in the general population and in pregnant women. Consequently, there has been a rise in frequency of neonatal abstinence syndrome (NAS), associated with buprenorphine use during pregnancy. The purpose of this study was to investigate correlations between buprenorphine and buprenorphine-metabolite concentrations in cord blood and onset of NAS in buprenorphine exposed newborns.

**Methods:**

Nineteen (19) newborns who met inclusion criteria were followed after birth until discharge in a double-blind non-intervention study, after maternal consent. Cord blood and tissue samples were collected and analyzed by liquid chromatography–mass spectrometry (LC–MS) for buprenorphine and metabolites. Simple and multiple logistic regressions were used to examine relationships between buprenorphine and buprenorphine metabolite concentrations in cord blood and onset of NAS, need for morphine therapy, and length of stay.

**Results:**

Each increase in 5 ng/ml level of norbuprenorphine in cord blood increases odds of requiring treatment by morphine 2.5 times. Each increase in 5 ng/ml of buprenorphine-glucuronide decreases odds of receiving morphine by 57.7 %. Along with concentration of buprenorphine metabolites, birth weight and gestational age also play important roles, but not maternal buprenorphine dose.

**Conclusions:**

LC–MS analysis of cord blood concentrations of buprenorphine and metabolites is an effective way to examine drug and metabolite levels in the infant at birth. Cord blood concentrations of the active norbuprenorphine metabolite and the inactive buprenorphine-glucuronide metabolite show promise in predicting necessity of treatment of NAS. These finding have implications in improving patient care and reducing healthcare costs if confirmed in a larger sample.

## Background

In recent years, there has been a rise in opioid-related drug use among pregnant women, specifically buprenorphine-containing medications (Eichel and Johannemann 2014). Buprenorphine is Food and Drug Administration (FDA)-approved for detoxification and maintenance therapy for opioid dependence, and is more frequently used in pregnancy than methadone due to better neonatal outcomes (Gugelmann and Nelson 2012; Jones et al. 2010; Prabhakar 2014). Maternal substance use disorder and development of neonatal abstinence syndrome (NAS) poses significant health problems for both woman and newborn with probable long term neurodevelopment adverse outcomes (Burns and Mattick 2007; Kellogg et al. 2011; SAMHSA 2010). From 1995 to 2009, cases of NAS have increased from 0.4 to 4.4 discharges per 1000 live births, representing what many feel is a national public health epidemic (Burns and Mattick 2007; Kellogg et al. 2011; SAMHSA 2010). Despite the rise in numbers for opioid-related NAS, predicting which infants will develop NAS following prenatal opioid exposure remains clinically difficult. Uncertainty of prediction of NAS requires all infants exposed to opioids during pregnancy to stay in the hospital for 5 days, which further increases cost of care. To that end, we investigated the possibility of drug-concentration and metabolite-concentration measurements as a predictor for the development and severity of NAS in exposed infants. In this study, we examine correlates of newborn cord blood buprenorphine and metabolite concentrations and the need to initiate opioid replacement therapy in the newborn, maternal buprenorphine dose, total morphine dose required for withdrawal, the duration of replacement therapy, and the length of neonatal intensive care unit (NICU) stay. Buprenorphine is metabolized by a Phase I oxidation to norbuprenorphine, which is a more potent mu-opioid agonist compared to buprenorphine, and Phase II metabolites include inactive glucuronide forms of buprenorphine and norbuprenorphine, all of which are monitored in our study (Oechsler and Skopp 2010). Additionally, we utilized a unique sample matrix, the newborn cord blood, in order to ensure measurable concentrations of the water-soluble metabolites. This work presents a newer approach for investigating predictors of NAS, and utilizes state-of-the-art analytical technology (LC–MS) with a sample matrix suitable for the quantification of drug metabolites.

## Methods

### Clinical sample collection

The protocol for this project was approved by our university’s Institutional Review Board (IRB), and represented a prospective, double-blind, non-interventional study. Women undergoing substance use disorder therapy were identified by history on presentation to the labor and delivery unit. Informed consent was obtained prior to delivery for those who met inclusion criteria for study. Inclusion criteria were gestational age more than 36 weeks, absence of congenital anomaly and no poly substance use. After delivery, cord blood was collected in ETDA–treated tubes and stored in a laboratory freezer (−20 °C) until analysis. Batches of samples were dispatched to the liquid chromatography–mass spectrometry (LC–MS) laboratory for quantification of buprenorphine and related metabolites. Analysts were blinded to maternal medication history. Cord tissue samples were also collected from all neonates and sent to a separate laboratory as per institutional guidelines. All neonates were then followed based on hospital protocol for the development of NAS, diagnosed using the Finnegan scoring system at 4 h intervals by trained nursing staff (Finnegan et al. 1975). Neonates with two Finnegan scores greater than 10, consecutively verified by separate nursing staff members, were transferred to the NICU, and started on morphine treatment. Morphine was started at 0.1 mg/kg every 4 h orally. Infants were weaned on doses of morphine at a rate of 10–20 % per day when Finnegan scores were below 7 for 24 h, and based on the infant’s clinical examination at the discretion of attending neonatologist. Morphine was discontinued when the dose reached less than 0.03 mg/kg per dose. Once weaned from morphine therapy, infants were monitored for 48 h prior to discharge. Results of cord blood metabolite were not available to clinical staff that cared for infants exposed to maternal drugs, but results of cord tissue were available.

### Analysis of cord blood samples

Cord blood samples were spiked with deuterium-labeled internal standards purchased from Cerilliant (RoundRock, TX). Samples were subjected to protein precipitation and solid-phase extraction (SPE) using Strata-X Drug B cartridges from Phenomenex (Torrance, CA). Chromatographic separation was carried out using a Phenomenex Kinetex C18 column (1.9 micron, 2.1 × 50 mm), and mass spectrometric detection was achieved using a Shimadzu LCMS–IT–TOF system operating in positive electrospray (ESI) mode (Columbia, MD). The LC–MS method utilized has been previously validated and published (Kyle et al. 2015).

### Statistical methods

Patient specific data were entered into a Microsoft Excel spreadsheet, cleaned, and thereafter imported into IBM SPSS Statistics version 20 (Armonk, NY) for analysis. Descriptive statistics were calculated for all variables. Simple logistic regressions were performed to assess the relationship between buprenorphine and metabolite cord blood concentrations and neonatal and maternal characteristics and necessity (yes/no) of morphine therapy. We converted odds ratios (OR) to effect size (ES) to find real associations, rather than *p* value to assess statistical significance, due to the study’s small sample size. Multiple logistic regressions were conducted for norbuprenorphine and buprenorphine-glucuronide cord blood concentration based on simple logistic regression.

## Results

### Patient population

A total of 19 women were enrolled for this study. Fifteen women were on Subutex™ (buprenorphine) and 4 were on Suboxone™ (buprenorphine and naloxone). All infants were from gestational age 36–41.2 weeks with mean of 38.5 weeks. Demographics of infants and their course and results from the LC–MS cord blood analysis are presented in Table 1. All women had history of smoking but no poly drug use.

**Table 1:** Sample descriptive statistics (N = 19)

Parameter	Mean (SD)
Duration of NICU stay (days)	6.5 (9.2)
Duration of morphine therapy (days)	5.1 (8.3)
Gestational age (weeks)	38.5 (1.4)
Birth weight (kg)	2.9 (0.39)
Maternal daily buprenorphine dose (mg)	11.2 (6.3)
Buprenorphine concentration (ng/ml)	9.0 (6.7)
Norbuprenorphine concentration (ng/ml)	13.9 (7.8)
Buprenorphine-glucuronide concentration (ng/ml)	14.0 (8.2)
Norbuprenorphine-glucuronide concentration (ng/ml)	23.3 (15.5)

### Cord blood concentrations and opioid therapy characteristics

Table 2 presents sample descriptive statistics and outcomes of simple logistic regression analyses examining relationships between buprenorphine and metabolite cord blood concentrations and neonatal and maternal characteristics, and necessity (yes/no) of morphine therapy. We converted odds ratios (OR) to effect size due to small sample size. Based on effect size, gestational age (4 weeks), birth weight (kg), norbuprenorphine concentration (5 ng/ml) and buprenorphine-glucuronide concentration (5 ng/ml) were associated with necessity (yes/no) of morphine therapy. Associations were strongest for birth weight (kg) and buprenorphine-glucuronide concentration (5 ng/ml). However, maternal daily buprenorphine dose, buprenorphine concentration (ng/ml) and norbuprenorphine-glucuronide concentration (ng/ml) were not associated with necessity of morphine therapy. For every increase of 1 week in gestational age from 36 to 40 weeks, the odds of necessity of morphine therapy increases 88 %. For every increase of one kg in birth weight, the odds of necessity increase 3.1 times. For every increase of 5 ng/ml in norbuprenorphine concentration, the odds of necessity increase 92 %. For every increase of 5 ng/ml in buprenorphine-glucuronide concentration, the odds of necessity decrease 54 %.

**Table 2:** Sample descriptive statistics and outcomes of simple logistic regression analyses examining relationships between buprenorphine and metabolite cord blood concentrations and neonate and maternal characteristics, and necessity (yes/no) of morphine replacement therapy^a^ (N = 19)

Parameter	Mean (SD)	Morphine replacement therapy necessity	
		OR (95 % CI)	Effect size
Duration of NICU stay (days)	6.6 (9.2)	NA	NA
Duration of morphine therapy (days)	5.1 (8.3)	NA	NA
Gestational age (weeks)	38.5 (1.4)	1.88 (0.12–29.78)	0.35
Birth weight (kg)	2.9 (0.39)	3.10 (0.23–42.65)	0.63
Maternal daily buprenorphine dose (mg)	11.2 (6.3)	1.01 (0.87–1.18)	0.01
Buprenorphine concentration (ng/ml)	9.0 (6.7)	0.97 (0.83–1.12)	0.01
Norbuprenorphine concentration (ng/ml)	13.8 (8.0)	1.92 (0.86–4.16)	0.36
Buprenorphine-glucuronide concentration (ng/ml)	14.0 (8.2)	0.46 (0.20–1.06)	0.43
Norbuprenorphine-glucuronide concentration (ng/ml)	23.3 (15.5)	0.98 (0.92–1.04)	0.01

*OR* odds ratio, *95* *% CI* 95 % confidence interval around odds ratio

^a^A statistically significant adjusted odds ratio greater than 1 indicates increased odds of morphine replacement therapy necessity/NICU transfer

As shown in Table 3, multiple logistic regressions were conducted for norbuprenorphine and buprenorphine-glucuronide concentration in cord blood. Maternal daily buprenorphine dose, cord blood buprenorphine and norbuprenorphine-glucuronide concentration were not included since they were not associated with morphine therapy necessity based on simple logistic regression modeling. Gestational age and birth weight were not selected for the final model since they are correlated. The adjusted odds ratios for norbuprenorphine is 2.504, which indicates that for every increase of 5 ng/ml in norbuprenorphine cord blood concentration the odds of morphine therapy need increases 2.5 times, controlling for buprenorphine-glucuronide. On the other hand, the adjusted OR for buprenorphine-glucuronide is 0.423, which indicates that for every increase of 5 ng/ml in buprenorphine-glucuronide, the odds of necessity decreases 57.7 % controlling for norbuprenorphine.

**Table 3:** Multiple logistic regression modeling of morphine replacement therapy necessity (yes/no) across norbuprenorphine and buprenorphine-glucuronide cord blood concentration

Variable	B coefficient	Standard error	aOR	95 % CI	Effect size
Norbuprenorphine (5 ng/ml)	0.918	0.575	2.504	0.812–7.726	0.507
Buprenorphine-glucuronide (5 ng/ml)	−0.860	0.448	0.423	0.176–1.019	0.475

*aOR* adjusted odds ratio, *95* *% CI* 95 % confidence interval around odds ratio

## Discussion

Fetal outcomes resulting from maternal opioid exposure depend on multiple variables, resulting in uncertainty of development of NAS in the newborn (Malek and Mattison 2011). Recent literature suggests 22–67 % infants present with NAS when prenatally there is a history of buprenorphine intake (Lacroix et al. 2011; Patel et al. 2013; Welle-Strand et al. 2012). In our study, that incidence is 36 % (7/19), which is in the range of what has been previously reported. However, the wide range of reported NAS incidence with buprenorphine use is indicative of the unpredictability of this syndrome following maternal use of buprenorphine. One factor that may contribute to this range includes variability in buprenorphine pharmacokinetics associated with CYP3A and CYP2C8, with heightened variation in CYP3A during pregnancy (Elkader and Sproule 2005; Lewis and Dinh 2015). Additionally, CYP2C8 is thought to be highly inducible, whereas the UGT isozymes responsible for Phase II metabolite formation may be less affected by pregnancy, thus implying a higher burden of active drugs with reduced conjugation (Isoherranen and Thummel 2013; Lewis and Dinh 2015). Metabolism within the placenta may also play a significant role in variations in fetal drug exposure to buprenorphine. Conversion of buprenorphine by aromatase (CYP19) to active norbuprenorphine increases with gestational age, thus supporting our results tying birth weight and gestational age to the development of NAS (Fokina et al. 2011), and CYP19 expression in syncytiotrophoblasts has been shown to be subject to genetic variation (Kumar and Mendelson 2011). Another possibility for explaining differences in NAS treatment needs lies in the individual infant’s expression of P-glycoprotein, an efflux transporter present in the placenta. Nekhayeva et al. (2006) demonstrated that this transporter is active against the parent drug, buprenorphine, among other xenobiotics. Finally, the impact of single nucleotide polymorphisms (SNPs) in opioid disposition genes has been investigated to reveal that variants in the OPRM1 118A>G and COMT 158A>G may be linked to lower NAS severity (Wachman et al. 2013).

To our knowledge, this is the largest study to date that has looked at umbilical cord plasma level of buprenorphine and its metabolites, to correlate these quantitative markers with neonatal course in NAS. Previous work by Conchiero and colleagues showed that buprenorphine metabolite concentrations could not predict the development of NAS (n = 5) (Concheiro et al. 2010a, b). Our data, although also limited by a small sample size, suggest that norbuprenorphine and the inactive glucuronide conjugate of buprenorphine can be useful in this respect. These data are in line with evidence presented in a review by Lewis et al., indicating a higher rate of active norbuprenorphine in infants of higher gestational age (Fokina et al. 2011; Lewis and Dinh 2015). Some investigators have suggested sex differences in NAS manifestation, with males showing NAS more often than females; however we were not able to explore this further due to our small sample size (O’Conner et al. 2013). We are in infancy of learning regarding genetic interplay of maternal, placental, and infant’s milieu with regard to maternal ingestion of opioid and resulting fetal effects. We propose that the investigation of metabolites in cord blood may be a practical way to study NAS in the buprenorphine exposed population, as these metabolites represent the end outcome of enzymatic and genetic variability in the maternal-placental-fetal triad.

Results from the MOTHER study (The Maternal Opioid Treatment: Human Experimental Research) show no relationship between maternal buprenorphine dose and the severity of NAS (Jones et al. 2012). Similar results were found in our study. No statistical significance was shown in regards to maternal buprenorphine dose at the time of delivery or the calculated cumulative dose throughout pregnancy with length of stay or duration of treatment required. Increasing birth weight and gestational age found to be strongly associated with development of NAS which can be explained by more conversion of buprenorphine to norbuprenorphine by aromatase by placental tissue, whose expression increases with gestational age from 16 to 24 km from 27–33 to 34–37 weeks (Fokina et al. 2011). Maternal history at delivery was taken as ‘evidence’ for maternal drug treatment, rather than followed by a confirmatory urine drug test. This discrepancy may have affected our results, as research has shown that ‘history of drug use’ is not the best marker for drug use in pregnancy (Ostrea et al. 2001).

Liquid-chromatography with mass spectrometric detection (LC–MS) has been successfully applied to the study of buprenorphine concentrations in urine, plasma, hair, sweat, and breast milk (Gray and Huestis 2007). Some groups have concentrated on pregnancy specific matrices such as umbilical cord tissue and meconium to quantify in utero exposure to these drugs (Kacinko et al. 2008a, b; Concherio et al. 2009; Concheiro-Guisan et al. 2009). The LC–MS method used in this study is adapted from these previous studies, all of which were supported by the National Institute on Drug Abuse (NIDA), a trusted source for drug analysis. Our method shows similar limits of quantification for buprenorphine, norbuprenorphine, and the glucuronide conjugates (Kyle et al. 2015). The importance of monitoring buprenorphine metabolites in this study cannot be underestimated. In a 2009 study by Kacinko et al. (2009), the glucuronidated version of norbuprenorphine was shown to be the primary urinary metabolite for pregnant women in their third trimester, indicating that the ability to measure this metabolite in the cord blood would be key to getting a full picture of neonatal exposure.

Recent study has suggested that cord tissue is equivalent and may be a superior matrix compared to meconium in detecting maternal drug exposure to cocaine, alcohol and amphetamines (Kacinko et al. 2008a, b). Our hospitals have utilized umbilical cord tissue for screening of maternal drug use as a routine and standard care when there is a history of maternal drugs or clinical suspicion based on maternal history or newborn’s course. Umbilical cord tissue gives only concentration of buprenorphine and norbuprenorphine when present but not buprenorphine-glucuronide and norbuprenorphine-glucuronide metabolite. Umbilical cord plasma is considered a relatively ‘new’ matrix useful for investigating in utero drug exposure, and limited head-to-head comparisons with cord tissue have been initiated (Gray and Huestis 2007) Both cord plasma and cord tissue have the advantage of being immediately available following delivery, and require a non-invasive collection (Gray and Huestis 2007). Our results indicate a false negative rate of 37 % using the cord tissue as the analytical matrix to examine the buprenorphine exposure, and of those “negative” samples, 57 % tested for norbuprenorphine-glucuronide as the highest opioid marker when the cord blood was analyzed. Only 63 % of neonates exposed to maternal buprenorphine in this study had positive cord tissue levels compared to 100 % having positive metabolite concentrations in cord blood. Other investigators have shown that the glucuronide conjugates can indeed be quantified in the cord tissue, but the drug concentrations are much lower than in cord blood and meconium, making this a much less sensitive matrix to monitor drug exposure to the neonate (Concheiro et al. 2010a, b). The lower sensitivity for cord tissue in monitoring glucuronide metabolites is likely related to the high water solubility of these conjugates, making them less likely to partition into tissue (Brunton et al. 2011). The institution’s cord tissue assay used in tandem with our study did not monitor for metabolite levels, and therefore, we found that LC–MS analysis of the cord blood with metabolite monitoring was superior to cord stat analysis without metabolite monitoring.

## Limitations

A limitation of our study is the small sample size (n = 19). It is unlikely to find significant associations between buprenorphine and buprenorphine-metabolite concentrations in cord blood and the need to initiate morphine replacement therapy in drug-exposed neonates at significance level α = 0.05 given such small sample size. According to statistical theory, p values related to ORs or regression coefficients depend on sample size. Large samples can lead to small p values without resulting in practical significance (e.g., statistical significance does not imply practical significance). On the other hand, small samples can lead to large p values without resulting in practical non-significance (e.g., statistical non-significance does not imply practical non-significance). Therefore, we used effect size (ES) converted from OR, rather than p values related to ORs or regression coefficients to assess the association between the dependent variable and those predictors since ES does not depend on sample size.

## Conclusions

In an examination of a small number of neonates with a history of in utero exposure to buprenorphine, we found a trend of an inverse relationship between cord blood concentrations of buprenorphine-glucuronide, and a positive relationship between cord blood concentrations of norbuprenorphine, which can help predict morphine treatment necessity along with birth weight and gestational age. Additionally, we found that glucuronide conjugate metabolite quantification via LC–MS in cord blood samples from these patients is essential in preventing false negative results that appear when only buprenorphine and norbuprenorphine are measured in cord tissue. This has implications for improving patient care along with significant reduction in patient care cost if proven in larger study.

## Abbreviations

CYP: cytochrome P450
EDTA: ethylenediaminetetraacetic acid
ES: effect size
ESI: electrospray
FDA: Food and Drug Administration
IRB: Institution Review Board
IT-TOF: ion trap–time-of-flight
LC–MS: liquid chromatography–mass spectrometry
MOTHER: Maternal Opioid Treatment: Human Experimental Research
NAS: neonatal abstinence syndrome
NICU: neonatal intensive care unit
NIDA: National Institute on Drug Abuse
OR: odds ratio
SNP: single nucleotide polymorphism
SPE: solid-phase extraction
